# Supplementary material for: Live slow-frozen human tumor tissues viable for 2D, 3D, ex vivo cultures and single-cell RNAseq
Source: Commun Biol. 2022 Oct 28;5:1144. doi: 10.1038/s42003-022-04025-0 (PMC9616892; doi:10.1038/s42003-022-04025-0)
Supplement: Supplementary file 2 — Supplementary Information [file 42003_2022_4025_MOESM2_ESM.pdf]

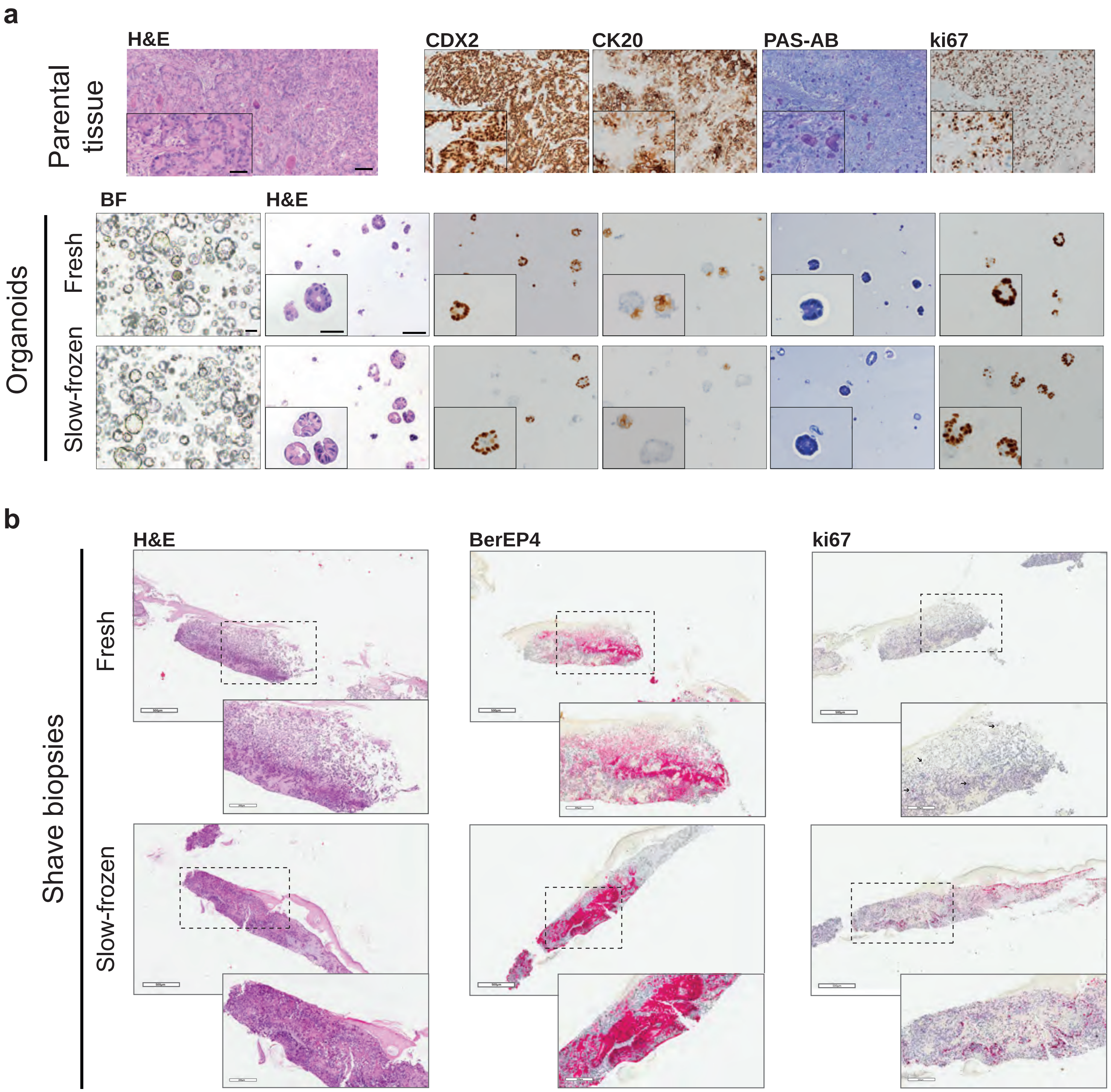
a
H&E
CDX2
CK20
PAS-AB
ki67
Parental tissue
BF
H&E
Organoids
Fresh
Slow-frozen
b
H&E
BerEP4
ki67
Shave biopsies
Fresh
Slow-frozen

Supplementary Figure 1: 3D and ex-vivo culture from fresh and slow-frozen matching tumor samples. a) Microscopic photographs of matched colon carcinoma liver metastasis parental tumor tissue and organoids derived from fresh and slow-frozen tissues. Scale bar lengths are 100μm, for insert microscopic photographs 50μm. CK20: Keratin 20, PAS-AB: Periodic acid-Schiff Alcian Blue; **b)** Microscopic photographs of shave biopsies from paired fresh or slow-frozen BCC stained with Hematoxylin-eosin (H&E) BerEp4 and Ki67 antibodies. Scale bar lengths are 500um, for insert microscopic photographs 200μm :

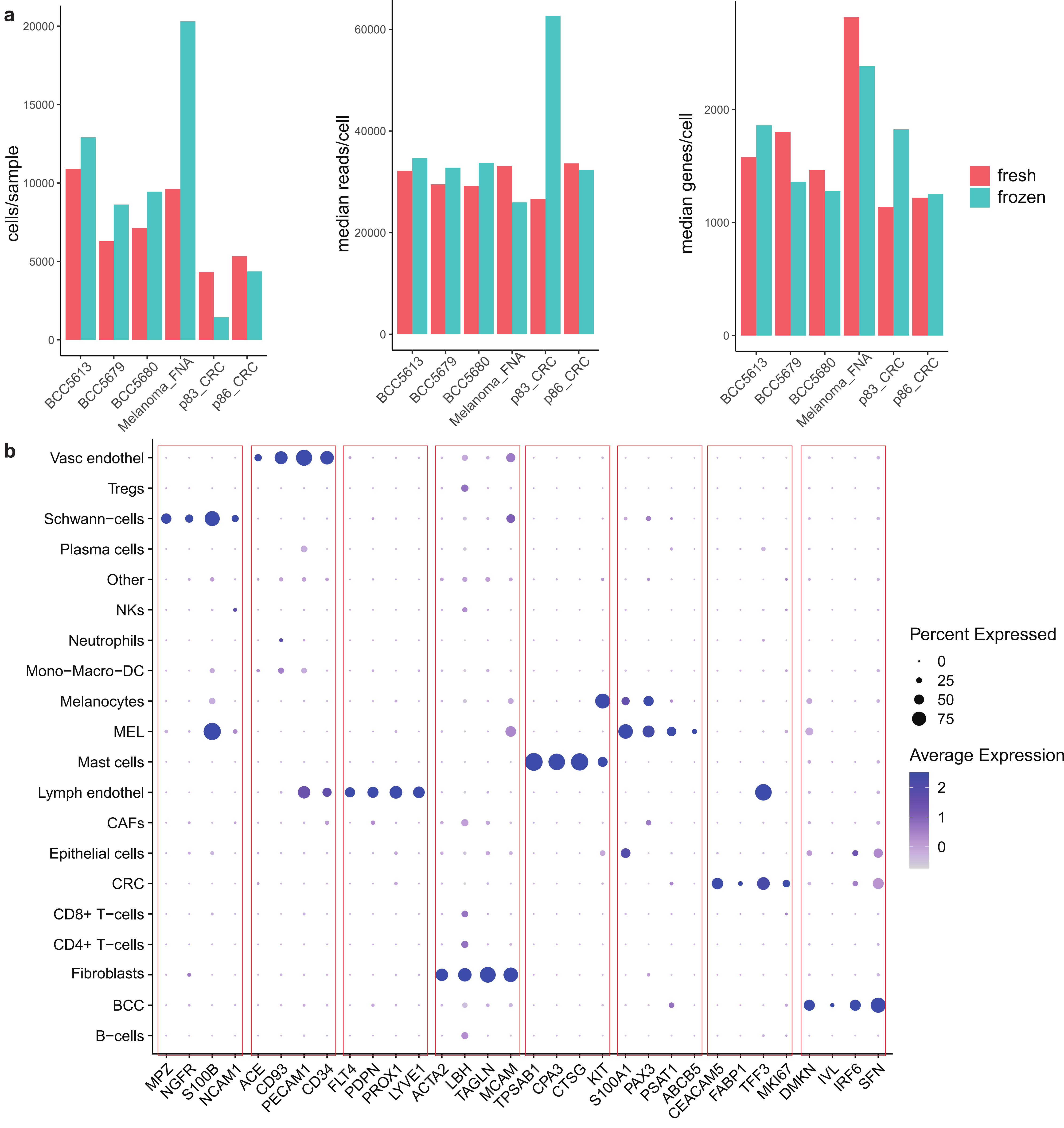
a
cells/sample
median reads/cell
median genes/cell
BCC5613
BCC5679
BCC5680
Melanoma_FNA
p83_CRC
p86_CRC
fresh
frozen
b
Vasc endothel
Tregs
Schwann−cells
Plasma cells
Other
NKs
Neutrophils
Mono−Macro−DC
Melanocytes
MEL
Mast cells
Lymph endothel
CAFs
Epithelial cells
CRC
CD8+ T−cells
CD4+ T−cells
Fibroblasts
BCC
B−cells
MPZ
NGFR
S100B
NCAM1
ACE
CD93
PECAM1
CD34
FLT4
PDPN
PROX1
LYVE1
ACTA2
LBH
TAGLN
MCAM
TPSAB1
CPA3
CTSG
KIT
S100A1
PAX3
PSAT1
ABCB5
CEACAM5
FABP1
TFF3
MKI67
DMKN
IVL
IRF6
SFN
Percent Expressed
0
25
50
75
Average Expression
2
1
0

Supplementary Figure 2: a) Quality control of the scRNAseq samples. **b)** Cell-type markers for Schwann-cells, vascular and lymphatic endothelial cells, CAFs, Mast cells, melanoma, CRC and BCC cells, the cell types, which were not called by SingleR.

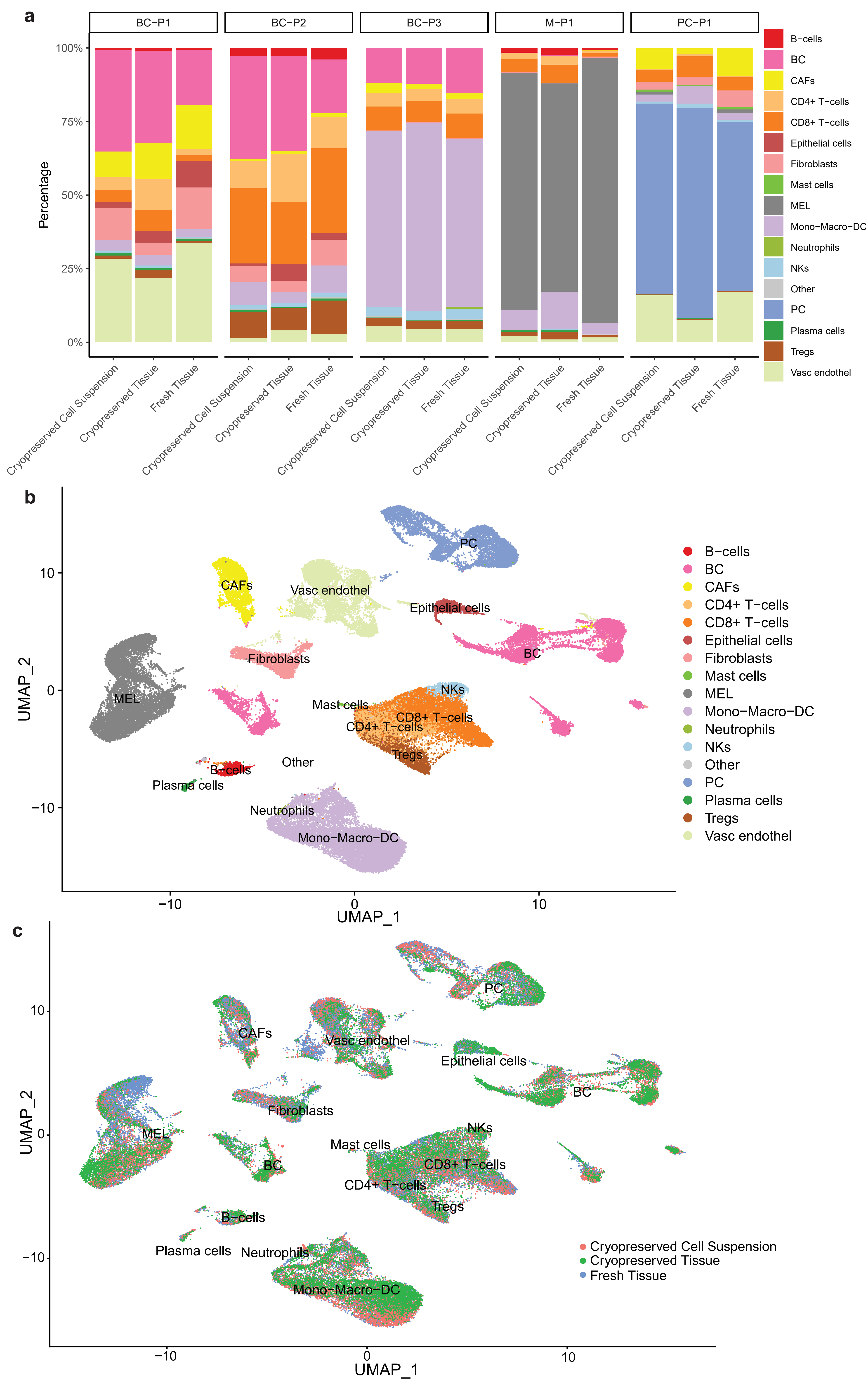
a
BC−P1
BC−P2
BC−P3
M−P1
PC−P1
Percentage
100%
75%
50%
25%
0%
Cryopreserved Cell Suspension
Cryopreserved Tissue
Fresh Tissue
B−cells
BC
CAFs
CD4+ T−cells
CD8+ T−cells
Epithelial cells
Fibroblasts
Mast cells
MEL
Mono−Macro−DC
Neutrophils
NKs
Other
PC
Plasma cells
Tregs
Vasc endothel
b
UMAP_2
UMAP_1
PC
CAFs
Vasc endothel
Epithelial cells
BC
Fibroblasts
MEL
NKs
Mast cells
CD8+ T−cells
CD4+ T−cells
Tregs
B−cells
Other
Plasma cells
Neutrophils
Mono−Macro−DC
c
Cryopreserved Cell Suspension
Cryopreserved Tissue
Fresh Tissue

Supplementary Figure 3: a) Cell type composition of the fresh and frozen tissue biopsy and cell suspension samples in the recent study published by Wu et al. (2021). Cell typing was performed using the cell typing of the current study. **b)** and **c)** UMAP representation of the cell types and sample treatment groups in the recent study published by Wu et al. (2021).

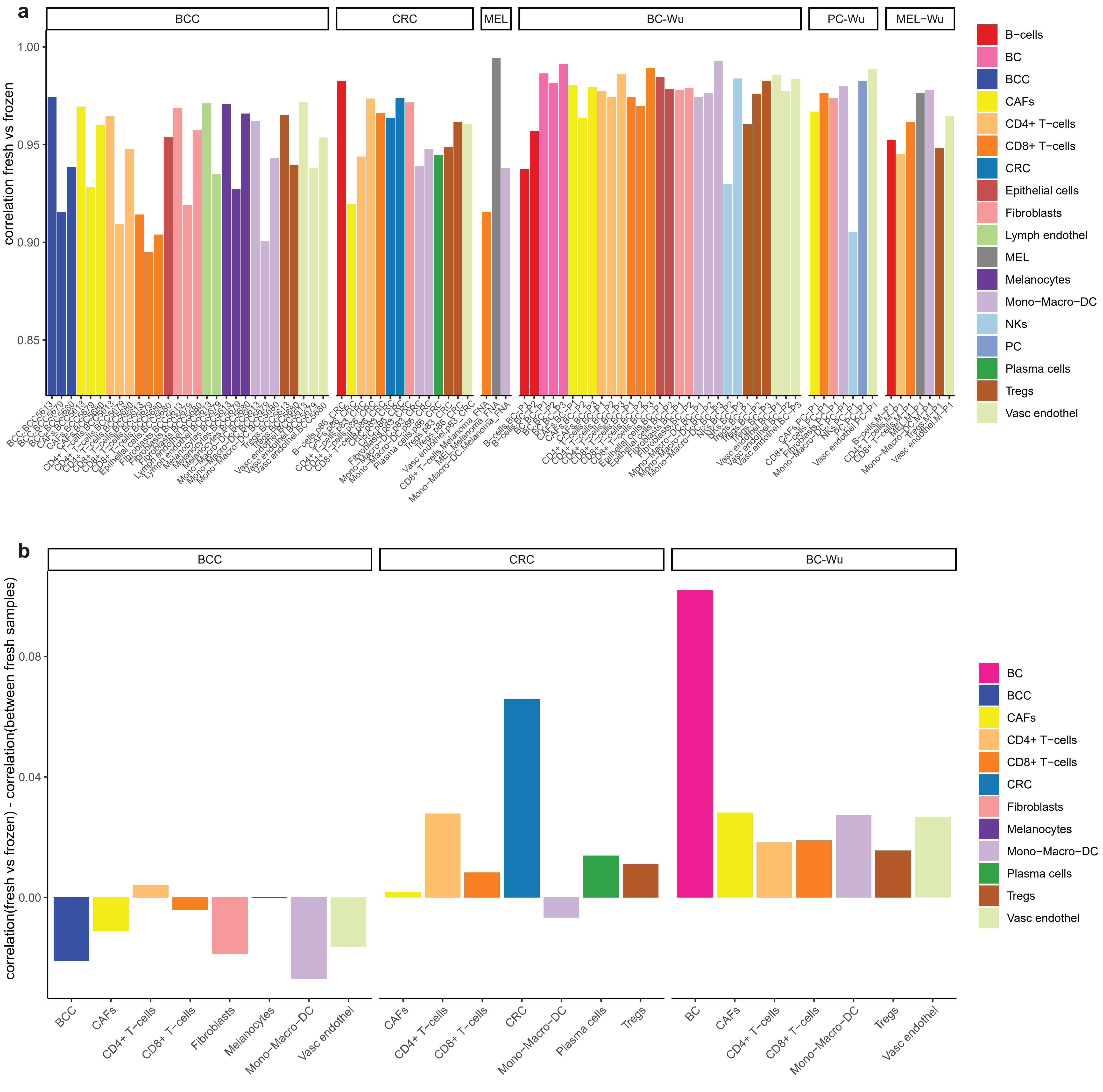
a
BCC
CRC
MEL
BC-Wu
PC-Wu
MEL-Wu
correlation fresh vs frozen
1.00
0.95
0.90
0.85
B−cells
BC
BCC
CAFs
CD4+ T−cells
CD8+ T−cells
CRC
Epithelial cells
Fibroblasts
Lymph endothel
MEL
Melanocytes
Mono−Macro−DC
NKs
PC
Plasma cells
Tregs
Vasc endothel
b
BCC
CRC
BC-Wu
correlation(fresh vs frozen) - correlation(between fresh samples)
0.08
0.04
0.00
BCC
CAFs
CD4+ T−cells
CD8+ T−cells
Fibroblasts
Melanocytes
Mono−Macro−DC
Vasc endothel
CAFs
CD4+ T−cells
CD8+ T−cells
CRC
Mono−Macro−DC
Plasma cells
Tregs
BC
CAFs
CD4+ T−cells
CD8+ T−cells
Mono−Macro−DC
Tregs
Vasc endothel
BC
BCC
CAFs
CD4+ T−cells
CD8+ T−cells
CRC
Fibroblasts
Melanocytes
Mono−Macro−DC
Plasma cells
Tregs
Vasc endothel

Supplementary Figure 4: a) Correlation coefficients measured between the average gene expressions of fresh and frozen cells for each cell type in each sample individually. **b)** The difference between the correlation coefficients measured between the average gene expressions of fresh cells from samples of different patients. Negative values indicate that the correlation between the gene expression of cells of two different patients is higher than the correlation measured between the gene expression of cells from a fresh and a frozen sample of one patient. The abbreviations BCC, CRC, MEL, BC-Wu, PC-Wu and MEL-Wu stand for basal cell carcinoma, colorectal carcinoma, melanoma, and breast cancer, prostate cancer and melanoma from the recent study of Wu et al. (2021).

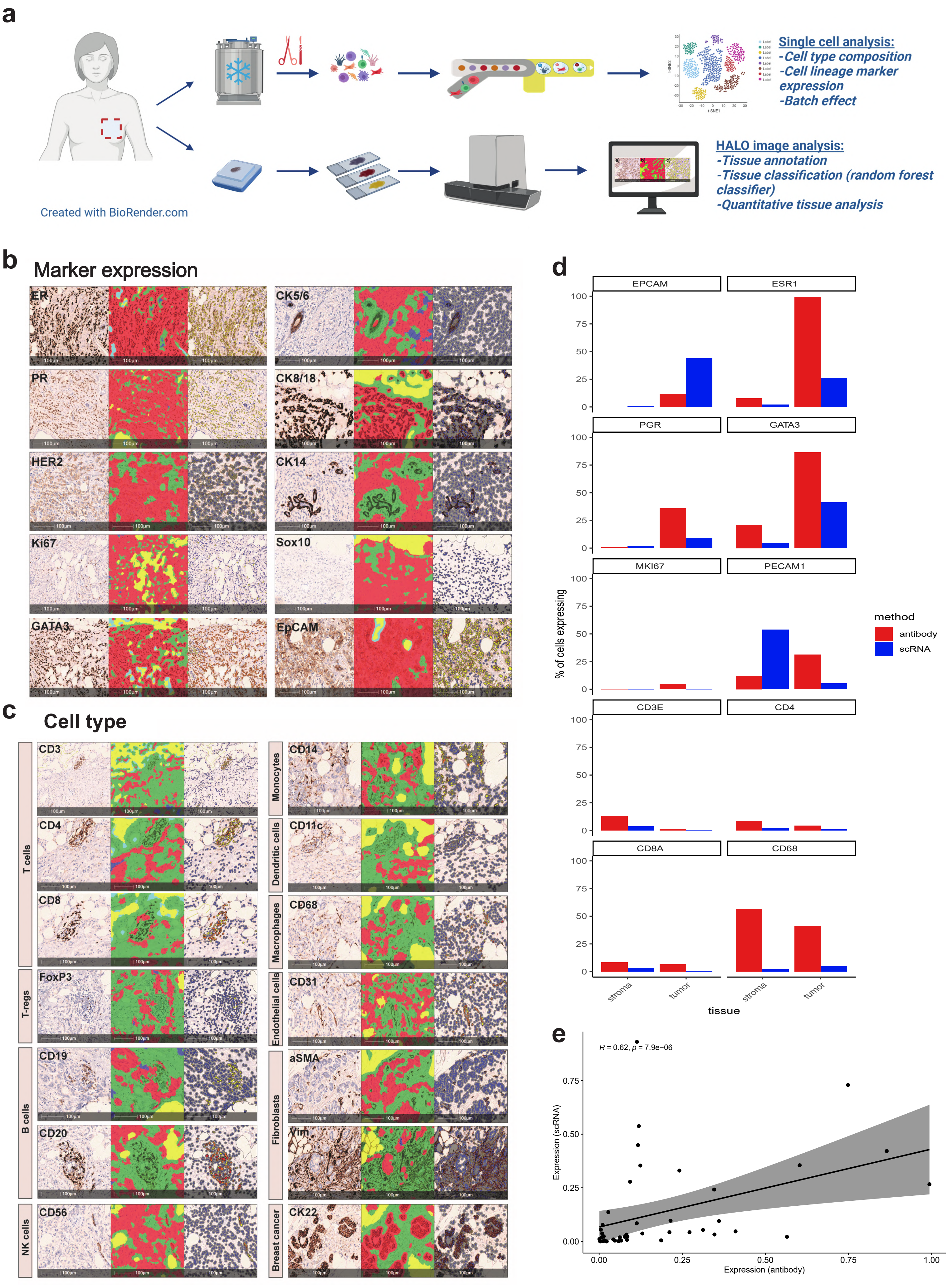
a
Single cell analysis:
-Cell type composition
-Cell lineage marker expression
-Batch effect
HALO image analysis:
-Tissue annotation
-Tissue classification (random forest classifier)
-Quantitative tissue analysis
Created with BioRender.com
b
Marker expression
ER
PR
HER2
Ki67
GATA3
CK5/6
CK8/18
CK14
Sox10
EpCAM
c
Cell type
T cells
CD3
CD4
CD8
T-regs
FoxP3
B cells
CD19
CD20
NK cells
CD56
Monocytes
CD14
Dendritic cells
CD11c
Macrophages
CD68
Endothelial cells
CD31
Fibroblasts
aSMA
Vim
Breast cancer
CK22
100µm
d
EPCAM
ESR1
PGR
GATA3
MKI67
PECAM1
CD3E
CD4
CD8A
CD68
% of cells expressing
method
antibody
scRNA
stroma
tumor
tissue
e
R = 0.62, p = 7.9e−06
Expression (scRNA)
Expression (antibody)
0.00
0.25
0.50
0.75
1.00

Supplementary Figure 5: a) Schematic representation of breast cancer tissue processing and analyses pipeline. Resected breast cancer tissues were split in two parts, one part was slow-frozen and then used for scRNAseq and the other part was preserved as a FFPE block and then used for IHC staining; **b)** and **c)** Analysis of marker expression **(b)** and cell type identification **(c)** by IHC staining on UHB129 tissue sections. left: representative images of indicated IHC stainings; middle: tissue classification (red: tumor cells, green: stroma, yellow: fat tissue, light blue: background); right: quantification of cells (nuclei) and indicated stainings using HALO imaging analysis software, scale bar: 100µm. **d)** Correlation of protein (quantified by HALO) and corresponding mRNA levels from scRNAseq. **e)** Correlation of mRNA and protein expression in stromal and tumor cells.

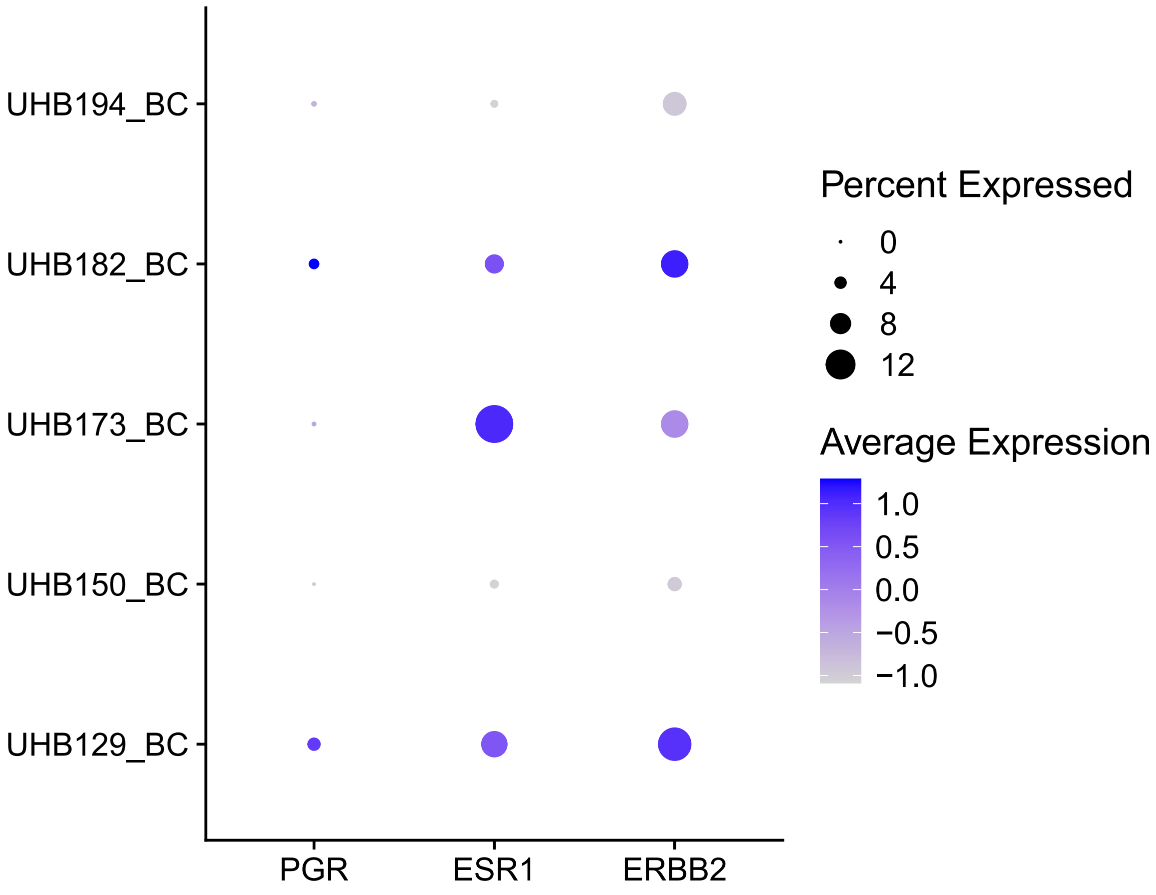

Supplementary Figure 6: Dotplot showing the expression of clinically relevant receptors in the five breast cancer tissues.

Supplementary Table 1: storage duration of slow-frozen samples per method

2D cultures

Melanoma slow-frozen biopsies	Successful	Not-successful
5-29days	4	1
1mo	6	6
2mo	4	2
3mo	5	1
4mo	0	1
5mo	1	1
6mo	0	2
7mo	2	0

3D and *ex vivo* cultures

Cancer type	Fresh processing	Slow-frozen processing
Colon metastasis in liver (P117)	07.10.2020	14.10.2020
Colon primary (P134)	12.11.2020	25.11.2022
Basal Cell Carcinoma (SB01)	12.05.2020	19.05.2020
Basal Cell Carcinoma (SB02)	01.07.2021	08.07.2021

scRNAseq application

Cancer type	Fresh processing	Slow-frozen processing
Basal Cell Carcinoma (BCC1) 5613	04.12.2019	05.02.2021

Basal Cell Carcinoma (BCC 2) 5679	06.12.2019	24.01.2020
Basal Cell Carcinoma (BCC 3)5680	06.12.2019	07.05.2020
Colon primary (CRC1)	16.07.2020	22.07.2020
Colon primary (CRC2) P86	18.07.2020	22.07.2020
Metastatic Melanoma (FNA)	22.01.2020	19.02.2020

Supplementary Table 2: Viability of cells isolated from fresh and slow-frozen samples

**Cancer type**	Sample type	Fresh		Slow frozen	
		Total cell number (per mL)	Viability (%)	Total cell number	Viability (%)
Basal Cell Carcinoma	BCC_5613	1.07×10^5 (1.07×10^6 cells/mL in 100µL)	88.6	6.6×10^4 (3.3×10^5 cells/mL in 200 µL)	64.6
Basal Cell Carcinoma	BCC_5679	1.15×10^5 (5.23×10^5 cells/mL in 220 µL	78.5	1.7×10^5 (8.85×10^5 cells/mL in 200 µL)	74.5
Basal Cell Carcinoma	BCC_5680	1.68×10^5 (1.12×10^6 cells/mL in 150 µL)<br><br>After DCR: 5×10^4 (10^6 cells/mL in 50 µL)	61.3 after DCR:77.5	6.3×10^4 (7.93×10^5 cells/mL in 80µL)	90.9
Colon primary	P83	2.5×10^6 (9.84×10^5 cells/mL in 3mL)	84	3.86×10^5 (3.86×10^5 cells/mL in 1mL)	81.3
Colon primary	P86	9.85×10^6 (1.97×10^6 cells/mL in 5 mL)	77.4	1.86×10^6 (1.24×10^6 cells/mL in 1.5mL)	81.1
Melanoma	Melanoma FNA	5×10^5 (3.7×10^6 cells /mL)	82.1	1.6×10^5 (4.15×10^5 cells/mL in 400µL)	82.5
Colon metastasis in liver	P117	8×10^6 (16×10^6 cells/mL in 2 mL)	51	2×10^6 (4×10^6 cells/mL in 2 mL)	50
Colon primary	P134	4×10^5 (4×10^5 cells/mL in 1 mL)	55	3×10^5 (3×10^5 cells/mL in 1 mL)	53

Supplementary Table 3: genes of the MelArray panel (for the mutation analysis of melanoma biopsies)

ABCB5	ACD	ACVR1C	AKAP9	AKT1	AKT2
AKT3	ALK	ANP32C	APC	ARID1A	ARID1B
ARID2	ARID4B	ARID5A	ASIP	ASPM	ATM
AURKA	AURKB	BAP1	BCL2	BCL2L12	BCLAF1
BRAF	BRCA1	BRCA2	CBL	CCND1	CCND2
CCND3	CDC42	CDK4	CDK6	CDKN1A	CDKN1B
CDKN2A	CDKN2C	CHD8	CTNNB1	CXCL1	CYP1B1
CYP7B1	DCT	DCUN1D3	DDR2	DDX3X	DLG1
DNMT1	DNMT3A	DNMT3B	DPP3	DYNC1I1	E2F1
EGFR	EIF1AX	EIF4A1	EP300	ERBB2	ERBB3
ETV6	EZH2	FAM58A	FANCA	FBXW7	FGFR1
FGFR2	FGFR3	FGFR4	FYN	GNA11	GNAI2
GNAQ	GNAS	HERC2	HLA-A	HLA-B	HLA-C
HRAS	IDH1	IGF2R	IQGAP1	ITGA5	JAK1
JAK2	JARID2	KDR	KIT	KMT2A	KMT2B
KMT2C	KMT2D	KNSTRN	KRAS	MAP2K1	MAP2K2
MAP2K4	MAP3K1	MAP3K2	MAP3K5	MAP3K8	MAP3K9
MAPK1	MAPK3	MC1R	MET	MITF	MLH1
MLH3	MTOR	MYCN	NF1	NFKBIE	NOTCH2
NRAS	NTRK1	OCA2	PARP1	PCDHGA1	PDGFRA
PIK3C2A	PIK3C3	PIK3CA	PIK3CB	PIK3R1	PIK3R4
PIKFYVE	PKD2	PLA2G6	PLCB1	PLCE1	PLEKHG4
PMEL	PMS2	POLQ	POT1	PPARG	PPP2R2A
PPP2R2B	PPP2R5C	PPP3CA	PPP6C	PRAME	PRKAR1A
PRKCD	PROS1	PTCH1	PTEN	PTPN11	PTPRF
PTPRJ	PTPRK	RAC1	RAD51B	RAF1	RASA1
RASA2	RASA3	RB1	RET	RICTOR	ROS1
RQCD1	SETD2	SF3B1	SHOC2	SLC1A4	SLC45A2
SMARCA4	SMO	SOS1	SOS2	SPRED1	SPRED2
SPRY4	SRC	STK11	TAOK1	TAOK2	TERF2
TERF2IP	TERT	TNRC6B	TP53	TRRAP	TSC1
TSC2	TUSC3	TYR	TYRP1		

Supplementary Table 4: primers used to confirm in the cell lines the mutations observed in the parental tumor

**Name of the primers**	**Primers-sequence**
**M13 cKIT Exon 9 F**	TGT AAA ACG ACG GCC AGT AAG TAT GCC ACA TCC CAA GT
**M13 cKIT Exon 9 R**	CAG GAA ACA GCT ATG ACC ATG GTC AAT GTT GGA ATG AA
**M13 cKIT Exon 11 F**	TGT AAA ACG ACG GCC AGT CCA GAG TGC TCT AAT GAC TGA
**M13 cKIT Exon 11 R**	CAG GAA ACA GCT ATG ACC GTT TCA GGT GGA ACA AAA CA
**M13 cKIT Exon 13 F**	TGT AAA ACG ACG GCC AGT CAT CAG TTT GCC AGT TGT G
**M13 cKIT Exon 13 R**	CAG GAA ACA GCT ATG ACC ATC TAG CAT TGC CAA AAT CA

**M13 cKIT Exon 17 F**	TGT AAA ACG ACG GCC AGT AAA AAG TTA GTT TTC ACT CTT TAC AA
**M13 cKIT Exon 17 R**	CAG GAA ACA GCT ATG ACC TCG AAA GTT GAA ACT AAA AAT CC
**M13 cKIT Exon 18 F**	TGT AAA ACG ACG GCC AGT GTA CTC AAG TTA TCA CTC CAC ATT T
**M13 cKIT Exon 18 R**	CAG GAA ACA GCT ATG ACC TCA AGA AGA TGC TCT GAG TCT AAT
**M13 GNAQ Exon 4 F**	TGT AAA ACG ACG GCC AGT CCG TAG ACA GCT TTG GTG TG
**M13 GNAQ Exon 4 R**	CAG GAA ACA GCT ATG ACC TTA CCA AAT GTA CTC AAG GCA TAA
**M13 GNAQ Exon 5 F**	TGT AAA ACG ACG GCC AGT TTT TCC CTA AGT TTG TAA GTA GTG C
**M13 GNAQ Exon 5 R**	CAG GAA ACA GCT ATG ACC CCC ACA CCC TAC TTT CTA TCA TTT AC
**M13 GNA11 Exon 4 F**	TGT AAA ACG ACG GCC AGT CTA CCT GAC CGA CGT TGA CC
**M13 GNA11 Exon 4 R**	CAG GAA ACA GCT ATG ACC GGC AAA TGA GCC TCT CAG TG
**M13 GNA11 Exon 5 F**	TGT AAA ACG ACG GCC AGT AGC CGA TGT CAG TCT GGT GT
**M13 GNA11 Exon 5 R**	CAG GAA ACA GCT ATG ACC AAA GGC AGA GGG AAT CAG AGG
**M13 BRAF-F Exon 15**	TGT AAA ACG ACG GCC AGT CTA AGA GGA AAG ATG AAG TAC TAT G
**M13 BRAF-R Exon 15**	CAG GAA ACA GCT ATG ACC CTA GTA ACT CAG CAG CAT CTC AG
**M13 NRAS2-F**	TGT AAA ACG ACG GCC AGT CCC CTT ACC CTC CAC AC
**M13 NRAS2-R**	CAG GAA ACA GCT ATG ACC AAC CTA AAA CCA ACT CTT CCC A
**M13 NRAS1-F**	TGT AAA ACG ACG GCC AGT GAT GTG GCT CGC CAA TTA AC
**M13 NRAS1-R**	CAG GAA ACA GCT ATG ACC TCC GAC AAG TGA GAG ACA GG
**PIK3CA-E9-F**	TGT AAA ACG ACG GCC AGT GCT AGA GAC AAT GAA TTA AGG GAA AA
**PIK3CA-E9-R**	CAG GAA ACA GCT ATG ACC CTC CAT TTT AGC ACT TAC CTG TGA C
**PIK3CA-Ex20-F**	TGT AAA ACG ACG GCC AGT CAT TTG CTC CAA ACT GAC CA
**PIK3CA-Ex20-R**	CAG GAA ACA GCT ATG ACC TGA GCT TTC ATT TTC TCA GTT ATC TTT TC
**PIK3CA_Ex9_F_2**	TGT AAA ACG ACG GCC AGT CAT CTG TGA ATC CAG AGG G
**PIK3CA_Ex9_R_2**	CAG GAA ACA GCT ATG ACC CTC CAT TTT AGC ACT TAC CTG TGA C
**PIK3CA_Ex20_F_2**	TGT AAA ACG ACG GCC AGT CAT CAT TTG CTC CAA ACT GAC
**PIK3CA_Ex20_R_2**	CAG GAA ACA GCT ATG ACC GAA AGC TCA CCT GGA TTC C
**PIK3CA Ex20_F_3**	TGT AAA ACG ACG GCC AGT CAT TTG AGC AAA GAC CTG AAG G
**PIK3CA Ex20_R_3**	CAG GAA ACA GCT ATG ACC TGA GCT TTC ATT TTC TCA GTT ATC
**BRAF_Ex11F**	TGT AAA ACG ACG GCC AGT TCC CTC TCA GGC ATA AGG TAA

**BRAF_Ex11R**	CAG GAA ACA GCT ATG ACC CGA ACA GTG AAT ATT TCC TTT GAT
**M13 KRAS Ex2 F_1**	TGT AAA ACG ACG GCC AGT AAG GCC TGC TGA AAA TGA C
**M13 KRAS Ex2 R_1**	CAG GAA ACA GCT ATG ACC TGG TCC TGC ACC AGT AAT ATG
**M13 KRAS Ex2 F_2**	TGT AAA ACG ACG GCC AGT AAG GCC TGC TGA AAA TGA CT
**M13 KRAS Ex2 R_2**	CAG GAA ACA GCT ATG ACC CTG GTG CAG GAC CAT TCT TTG A
**M13 HRAS Ex 1_Fwd**	TGT AAA ACG ACG GCC AGT CAGGAGACCCTGTAGGAGGA
**M13 HRAS Ex 1_Rev**	CAG GAA ACA GCT ATG ACC CCTATCCTGGCTGTGTCCTG
**M13 HRAS Ex 2_Fwd**	TGT AAA ACG ACG GCC AGT AGAGGCTGGCTGTGTGAACT
**M13 HRAS Ex 2_Rev**	CAG GAA ACA GCT ATG ACC TCACGGGGTTCACCTGTACT
**M13 HRAS Ex 3_Fwd**	TGT AAA ACG ACG GCC AGT CTGTCCTCTCTGCGCATGTC
**M13 HRAS Ex 3_Rev**	CAG GAA ACA GCT ATG ACC AGGGTCAGTGAGTGCTGCTC
**M13 HRAS Ex 4_Fwd**	TGT AAA ACG ACG GCC AGT ACAGCAGGGAGCCCCTCAC
**M13 HRAS Ex 4_Rev**	CAG GAA ACA GCT ATG ACC CATGTCCTGAGCTTGTGCTG

Supplementary Table 5: Cell type merging

Merged cell type calls	Original SingleR using Blueprint+ENCODE
Adipocytes	Adipocytes
Other	Astrocytes
CD4+ T-cells	CD4+ T-cells
CD4+ T-cells	CD4+ Tcm
CD4+ T-cells	CD4+ Tem
CD8+ T-cells	CD8+ T-cells
CD8+ T-cells	CD8+ Tcm
CD8+ T-cells	CD8+ Tem
Other	Chondrocytes
B-cells	Class-switched memory B-cells
Other	CLP
Other	CMP
Mono-Macro-DC	DC
Endothelial cells	Endothelial cells
Other	Eosinophils
Epithelial cells	Epithelial cells
Other	Erythrocytes
Fibroblasts	Fibroblasts
Other	GMP
Other	HSC
Epithelial cells	Keratinocytes
Mono-Macro-DC	Macrophages

Mono-Macro-DC	Macrophages M1
Mono-Macro-DC	Macrophages M2
Other	Megakaryocytes
Melanocytes	Melanocytes
B-cells	Memory B-cells
Other	MEP
Other	Mesangial cells
Mono-Macro-DC	Monocytes
Other	MPP
Endothelial cells	mv Endothelial cells
Other	Myocytes
B-cells	naive B-cells
Other	Neurons
Other	Neutrophils
NKs	NK cells
Other	Pericytes
Plasma cells	Plasma cells
Other	Preadipocytes
Other	Skeletal muscle
Other	Smooth muscle
Tregs	Tregs
