# Supplementary material for: Live slow-frozen human tumor tissues viable for 2D, 3D, ex vivo cultures and single-cell RNAseq
Source: Commun Biol. 2022 Oct 28;5:1144. doi: 10.1038/s42003-022-04025-0 (PMC9616892; doi:10.1038/s42003-022-04025-0)
Supplement: Supplementary file 3 — Description of Additional Supplementary Files [file 42003_2022_4025_MOESM3_ESM.pdf]

Description of Additional Supplementary Files

File name: Supplementary Data 1
Description: This table contains the Reactome pathways collected using the msigdbr package (v7.4.1) and their normalized enrichment scores (fresh vs slow-frozen cells) for each disease and cell type calculated by fgsea (v1.20.0). The pathways are ordered based on the median rank of most enriched pathways in across the cell types.
