# Supplementary material for: Live slow-frozen human tumor tissues viable for 2D, 3D, ex vivo cultures and single-cell RNAseq
Source: Commun Biol. 2022 Oct 28;5:1144. doi: 10.1038/s42003-022-04025-0 (PMC9616892; doi:10.1038/s42003-022-04025-0)
Supplement: Supplementary file 5 — Reporting Summary [file 42003_2022_4025_MOESM5_ESM.pdf]

Corresponding author(s): Mitchell P. Levesque

Last updated by author(s): Aug 26, 2022

Reporting Summary

Nature Research wishes to improve the reproducibility of the work that we publish. This form provides structure for consistency and transparency in reporting. For further information on Nature Research policies, see our Editorial Policies and the Editorial Policy Checklist.

Statistics

For all statistical analyses, confirm that the following items are present in the figure legend, table legend, main text, or Methods section.

n/a	Confirmed	
☐	☒	The exact sample size (*n*) for each experimental group/condition, given as a discrete number and unit of measurement
☐	☒	A statement on whether measurements were taken from distinct samples or whether the same sample was measured repeatedly
☐	☒	The statistical test(s) used AND whether they are one- or two-sided<br>*Only common tests should be described solely by name; describe more complex techniques in the Methods section.*
☐	☒	A description of all covariates tested
☐	☒	A description of any assumptions or corrections, such as tests of normality and adjustment for multiple comparisons
☐	☒	A full description of the statistical parameters including central tendency (e.g. means) or other basic estimates (e.g. regression coefficient) AND variation (e.g. standard deviation) or associated estimates of uncertainty (e.g. confidence intervals)
☐	☒	For null hypothesis testing, the test statistic (e.g. *F*, *t*, *r*) with confidence intervals, effect sizes, degrees of freedom and *P* value noted<br>*Give P values as exact values whenever suitable.*
☒	☐	For Bayesian analysis, information on the choice of priors and Markov chain Monte Carlo settings
☒	☐	For hierarchical and complex designs, identification of the appropriate level for tests and full reporting of outcomes
☒	☐	Estimates of effect sizes (e.g. Cohen's *d*, Pearson's *r*), indicating how they were calculated

Our web collection on statistics for biologists contains articles on many of the points above.

Software and code

Policy information about availability of computer code

Data collection	CellRanger 3.1 developed by 10xGenomics was used to analyze raw scRNA sequencing files.
Data analysis	We used custom data analysis scripts described at: https://github.com/uzh-dqbm-cmi/lymphoma_tvec_study<br>The stainings made on breast cancer were analysed by the HALO software which is a standard image analysis platform for quantitative tissue analysis in digital pathology.

For manuscripts utilizing custom algorithms or software that are central to the research but not yet described in published literature, software must be made available to editors and reviewers. We strongly encourage code deposition in a community repository (e.g. GitHub). See the Nature Research guidelines for submitting code & software for further information.

Data

Policy information about availability of data

All manuscripts must include a data availability statement. This statement should provide the following information, where applicable:

- Accession codes, unique identifiers, or web links for publicly available datasets
- A list of figures that have associated raw data
- A description of any restrictions on data availability

The scRNA sequencing data generated in this study has been deposited to the European Genome-phenome Archive (EGA) under the study accession EGAS00001005891.
We used publicly available dataset from the work of Wu et al: Cryopreservation of human cancers conserves tumour heterogeneity for single-cell multi-omics analysis. Genome Med. 2021 May 10;13(1):81. (EGA accession code EGAS00001005115)

Field-specific reporting

Please select the one below that is the best fit for your research. If you are not sure, read the appropriate sections before making your selection.

☒ Life sciences ☐ Behavioural & social sciences ☐ Ecological, evolutionary & environmental sciences

For a reference copy of the document with all sections, see nature.com/documents/nr-reporting-summary-flat.pdf

Life sciences study design

All studies must disclose on these points even when the disclosure is negative.

Sample size	No power-calculations were performed. The explorative nature of the study did not allow for estimating effect sizes prior to the analysis.
Data exclusions	No data were excluded from the analysis.
Replication	Multiple patients' samples were used for the colorectal cancer, breast cancer and basal cell carcinoma to give statistical power to our investigations.
Randomization	Tissue samples were processed, divided into equal parts and randomly assigned either to be processed fresh or after freezing.
Blinding	The experimental set-up did not make blinding possible.

Reporting for specific materials, systems and methods

We require information from authors about some types of materials, experimental systems and methods used in many studies. Here, indicate whether each material, system or method listed is relevant to your study. If you are not sure if a list item applies to your research, read the appropriate section before selecting a response.

Materials & experimental systems

n/a	Involved in the study	
☐	☒	Antibodies
☐	☒	Eukaryotic cell lines
☒	☐	Palaeontology and archaeology
☒	☐	Animals and other organisms
☒	☐	Human research participants
☐	☒	Clinical data
☒	☐	Dual use research of concern

Methods

n/a	Involved in the study	
☒	☐	ChIP-seq
☒	☐	Flow cytometry
☒	☐	MRI-based neuroimaging

Antibodies

Antibodies used	Ki-67 (Dako Cat. No. IR626), CDX2 (Ventana Cat. No. 760-4380) and CK20 (Ventana Cat. No. 790-4431).<br>CD11c (Cellmarque, 111M-18); CD56 (Ventana, 790-4465); CD14 (Ventana, 760-4523); CD68 (Dako, IR613); CD19 (Dako, IR656); CD8 (Ventana, 790-4460); CD3 (Ventana, 790-4341); CK14 (Ventana, 760-4805); CD31 (Ventana, 760-4378); CK22 (Immuno, Bio MM-1012-02); CD4 (Ventana, 790-4423); CK5/6 (Ventana, 790-4554); EpCAM (Ventana, 760-4383); CK8 (Ventana, 760-2637); ER (Ventana, 790-4324); HER2 (Ventana, 790-2991); FoxP3 (Abcam, ab99963); Ki67 (Dako, IR626); Gata3 (Ventana, 760-4897); PR (Ventana, 790-2223); SMA (Ventana, 760-2833); Vimentin (Ventana, 790-2917); Sox10 (Ventana, 760-4968); Ki67 (DAKO, M7240) and BerEP4 (Dako. M0804)
Validation	All antibodies are used in routine pathology departments

Eukaryotic cell lines

Policy information about cell lines

Cell line source(s)	The melanoma cell lines were produced in our laboratory from surplus surgically derived melanoma material
Authentication	All cell lines have been tested for their mutational state.
Mycoplasma contamination	The cells tested negative for mycoplasma
Commonly misidentified lines (See ICLAC register)	*Name any commonly misidentified cell lines used in the study and provide a rationale for their use.*

Clinical data

Policy information about clinical studies

All manuscripts should comply with the ICMJE guidelines for publication of clinical research and a completed CONSORT checklist must be included with all submissions.

Clinical trial registration	*Provide the trial registration number from ClinicalTrials.gov or an equivalent agency.*
Study protocol	KEK 2017-00688 (Ethics commission Zürich), EKBB 2019-00816 (Ethics commission Basel), EKNZ 2018-00729 (Ethics commission Basel). Only surplus material from routine surgeries were used in this study.
Data collection	Patient and clinical data were anonymised immediately after sample collection. Only basic clinical information were used in this study (age, sex, location of the sample and type of tumor)
Outcomes	*Describe how you pre-defined primary and secondary outcome measures and how you assessed these measures.*
